# Treatment Options for Colistin Resistant *Klebsiella pneumoniae*: Present and Future

**DOI:** 10.3390/jcm8070934

**Published:** 2019-06-28

**Authors:** Nicola Petrosillo, Fabrizio Taglietti, Guido Granata

**Affiliations:** Systemic and Immunocompromised Host Infection Unit, National Institute for Infectious Diseases “L. Spallanzani”, IRCCS–Via Portuense, 292 00149 Rome, Italy

**Keywords:** *Klebsiella pneumoniae*, colistin-resistant, cefiderocol, ceftazidime/avibactam, colistin, fosfomycin, plazomicin, tigecyclin

## Abstract

Multidrug-resistant (MDR) *Klebsiella pneumoniae* represents an increasing threat to human health, causing difficult-to-treat infections with a high mortality rate. Since colistin is one of the few treatment options for carbapenem-resistant *K. pneumoniae infections*, colistin resistance represents a challenge due to the limited range of potentially available effective antimicrobials, including tigecycline, gentamicin, fosfomycin and ceftazidime/avibactam. Moreover, the choice of these antimicrobials depends on their pharmacokinetics/pharmacodynamics properties, the site of infection and the susceptibility profile of the isolated strain, and is sometimes hampered by side effects. This review describes the features of colistin resistance in *K. pneumoniae* and the characteristics of the currently available antimicrobials for colistin-resistant MDR *K. pneumoniae*, as well as the characteristics of novel antimicrobial options, such as the soon-to-be commercially available plazomicin and cefiderocol. Finally, we consider the future use of innovative therapeutic strategies in development, including bacteriophages therapy and monoclonal antibodies.

## 1. Introduction

The global rise of multidrug-resistant (MDR) gram-negative bacteria represents an increasing threat to human health [1,2,3]. Among gram-negative bacteria, the rod-shaped bacterium *K. pneumoniae* is one of the commonest cause of healthcare associated infection in humans including urinary tract infections, pneumonia, meningitis and sepsis [1,2,3,4]. *K. pneumoniae* is also the most common multidrug-resistant and carbapenem-resistant pathogen in the healthcare setting [1,2,3,4]. During the last decades, polymyxins have represented the most used antimicrobial options against carbapenem-resistant *K. pneumoniae*. Indeed, polymyxin E (colistin) has been considered as a “last resort” antimicrobial to fight MDR *K. pneumoniae* infections, often representing the only antimicrobial to achieve adequate serum levels and minimum inhibitory concentrations (MIC) [5]. Therefore, the recent reports of colistin-resistant *K. pneumoniae* isolates raises concern, considering the further limitations of antimicrobial options and the high mortality rate associated with these infections [6]. We describe the currently available therapeutic options and the future approaches in development for the treatment of colistin-, carbapenem-resistant *K. pneumoniae* (C-C-RKp) infections.

### Colistin Resistance in K. pneumoniae

Colistin (Polymyxin E) is a cyclic polypeptide bactericidal antimicrobial of the polymyxin class, possessing targeted Gram-negative activity. Colistin chemical structure resembles that of other antimicrobial peptides produced by eukaryotic cells, such as defensins, and its peculiar tridimensional structure provides at least three different mechanisms of antimicrobial action [7,8,9].

First, due to its chemical structure, colistin represents a potent amphipathic agent and acts in a detergent-like fashion to disrupt the structure of the outer membrane of Gram-negative bacteria. More precisely, the electrostatic interaction between this antimicrobial and the anionic phosphate group of the lipopolysaccharide leads to the displacement of divalent cations, such as calcium and magnesium, from the negatively charged phosphate groups of the bacterial membrane [10]. The subsequent destabilization of bacterial membrane causes cellular contents’ leakage and, ultimately, bacterial lysis and death [10].

Second, colistin directly binds and neutralizes the “lipid A” portion of the bacterial lipopolysaccharides, contributing to bacterial cell lysis [11]. Third, a colistin-mediated inhibition of vital respiratory enzymes located in the bacterial inner membrane has been described [12].

Despite its potent bactericidal activity, colistin use is often associated with relevant side effects, including nephrotoxicity and neurotoxicity, that have been reported in 14–53% and 4–6% of patients, respectively [13,14,15]. The exact mechanisms causing these adverse events are not well understood but may be explained by colistin hydrophobic properties [8,16].

Until recently, colistin was considered as a “last resort” antimicrobial to treat infections due to carbapenem-resistant *K. pneumoniae* infections. Unfortunately, with the increase in use of colistin, the presence of colistin-resistant *K. pneumoniae* has been reported. The European Committee on Antimicrobial Susceptibility Testing (EUCAST) defined in vitro colistin resistance for *K. pneumoniae* as a minimal inhibitory concentration (MIC) of >2 mg/L, recommending performing the colistin MIC determination with broth microdilution [17].

During the last decade, the rate of colistin resistance among carbapenem-resistant *K. pneumoniae* progressively increased from less than 2% to 9% worldwide [18,19,20,21,22]. In Europe, since 2013 colistin resistance rate increased up to one-third of carbapenem-resistant isolates [23]. In addition, multiple outbreaks of colistin-resistant *K. pneumoniae* have been reported in USA [24], Canada [25], South America [26] and Europe [19,27,28,29,30]. Recent reports evidence even more concerning data in some European countries including Italy, Greece, Spain, Hungary, with resistance to colistin up to 43% of carbapenem-resistant *K. pneumoniae* in Italy, 20.8% in Greece and up to 31% in Spain [31,32,33].

Interestingly, colistin resistance in *K. pneumoniae* is mediated by several mechanisms. The most common mechanism is the modification in the molecular structure of the bacterial lipopolysaccharides, mediated by cationic substitutions altering the electrostatic interaction between colistin and the lipopolysaccharide itself [8]. These lipopolysaccharides modifications are mediated by genetic mutations on chromosomal genes, such as amino acids’ substitutions, insertions or deletions. Additionally, the acquisition of plasmidic genes can confer colistin resistance [34,35]. The plasmidic gene mcr-1, firstly described in China in 2011, is the main cause of plasmidic-mediated colistin resistance worldwide [35,36,37,38,39]. The mcr-1 plasmid codes for a phosphoethanolamine transferase enzyme which leads to the addition of phosphoethanolamine in the bacterial lipopolysaccharide structure, altering its electrostatic charge and therefore reducing the affinity with colistin. Beside mcr-1 gene, other mcr homologs (i.e., mcr-1, mcr-3, mcr-7 and mcr-8) have been reported in *K. pneumoniae* [40,41,42,43].

The emergence of a transmissible, plasmid-mediated colistin resistance is particularly alarming, because it may accelerate the spread of colistin resistance among different *K. pneumoniae* strains and among different bacteria [44,45].

Regarding the occurrence of colistin resistance in *K. pneumoniae,* several hypotheses are reported in the literature [46]. The most plausible one is that colistin resistance develops under antibiotic treatment with colistin [37,46,47]. A correlation between national antimicrobial consumption levels and resistance development has been recently confirmed [48].

Finally, there is evidence that infections due to colistin-resistant *K. pneumoniae* have higher mortality rates than those caused by colistin susceptible strains [6,49]. This can be explained by delay in detection of colistin resistance, and by the low activity and pharmacokinetic weakness of some available treatment options [50]. In an outbreak investigation carried out in 2016 in Turkey in a 550-bed tertiary care hospital, all five cases of infection due to colistin-resistant *K. pneumoniae* died, with a 14-day mortality rate of 60% and a 30-day mortality rate of 100% [51]. These findings were confirmed by a large prospective cohort study performed in 2015–2016 including 115 patients diagnosed with colistin-resistant *K. pneumoniae* infections. The study reported an overall 30 days mortality rate of 61% [52]. In 2011, in an Italian prospective study on patients with MDR *K. pneumoniae* one of the variable independently associated with higher mortality was infection due to a colistin-resistant *K. pneumoniae* strain (Odds Ratio (OR): 95% Confidence interval (CI): 4.14; 1.17–14.74 *p* < 0.02) [6].

## 2. Currently Available Therapeutical Options against C-C-RKp

The superiority of antimicrobial combination in comparison to monotherapy for the treatment of infections due to MDR *K. pneumoniae* has yet to be fully elucidated. Retrospective studies report lower mortality rates with combination therapy [53,54]. For infections due to carbapenem-resistant *K. pneumoniae* the use of carbapenem, generally meropenem, should also be considered when carbapenem MIC is ≤16 mg/L, at a double dosage and prolonged infusion [55]. Conversely, carbapenem MICs higher than 16 mg/L do not warrant the use of meropenem.

Resistance to colistin makes the choice of antimicrobials more difficult. Indeed, few antimicrobial options are currently available for Colistin-, Carbapenem-Resistant Klebsiella pneumoniae (C-C-RKp) infections (Table 1). Their use mainly depends on the antimicrobial susceptibility profile of the isolate, the site of infection, their pharmacokinetics/pharmacodynamics (PK/PD) properties and their potential adverse events. The best available options are discussed below. In Table 2, we discuss possible antimicrobial combinations, according to the site of infection and the MIC value for meropenem.

### 2.1. Fosfomycin

Fosfomycin is a broad-spectrum antimicrobial derived from phosphonic acid, with time-dependent bactericidal activity against both Gram-positive and Gram-negative pathogens. It acts by inhibiting the enzyme involved in peptidoglycan synthesis, the main component of the bacterial wall [59,79].

More precisely, after penetration into the cell wall through the alpha-glycerol-phosphate and glucose-6-phosphate transport system, fosfomycin impairs the formation of N-acetylmuramic acid leading to bacterial death [59]. This antimicrobial is therefore active against a wide range of bacteria, including *Staphylococcus aureus*, coagulase negative *Staphylococcus* spp., Enterococci, *Haemophilus* spp., and Enterobacteriaceae [56].

Fosfomycin possesses structural simplicity and low molecular weight and has excellent safety and tolerability profile; of note, it does not cause nephrotoxicity or hepatotoxicity. However, it should be considered that a high amount of sodium is administered with every gram of fosfomycin and therefore, during a treatment with fosfomycin, a frequent evaluation of serum electrolytes is suggested [57,60].

Due to its broad-spectrum activity and its pharmacokinetics/pharmacodynamics profile, fosfomycin can be considered for the treatment of infections involving different body sites, including pneumonia, osteomyelitis, meningitis, surgical infections, abdominal infections and bloodstream infections [60].

Fosfomycin is currently available as oral formulation (i.e., fosfomycin tromethamine) and as disodic fosfomycin for intravenous administration [57,58,60]. While the oral administration of fosfomycin tromethamine offers a bioavailability higher than 50% and is of particular interest in the treatment of uncomplicated urinary tract infections, the treatment of systemic infections requires an intravenous route, at a dosage of 6 g every 6 h (24 g administered daily) or more. To note, the average serum half-life of fosfomycin is 2 h, but can be lower in critical patients or patients in septic shock, depending on renal function [57,58,60]. For the treatment of patients with creatinine clearance lower than 30 mL/min, fosfomycin dosage should be reduced to 4 g every 8 h [57,58,60].

A crucial open issue for MDR *K. pneumoniae* treatment with fosfomycin is the in vitro sensibility assay interpretation [80]. In comparison with the agar dilution methods, which is the gold standard for fosfomycin resistance evaluation in *K. pneumoniae*, high rates of false resistance have been reported adopting an automated test. On the other hand, high rates of false susceptibility results have been reported adopting the Etest [80,81,82]. Therefore, automated susceptibility tests for fosfomycin resistance evaluation are not completely reliable, and the MIC provided by in vitro tests does not represent a predictive factor for the development of resistance in vivo [80,81,82].

Additionally, from the clinician perspective, a crucial aspect is that monotherapy with fosfomycin may select resistance in *K. pneumoniae* [79,83,84]. This aspect is of particular concern, as fosfomycin resistance can be mediated through plasmids that code for fosfomycin-inactivating enzymes (fosA) [83]. According to these considerations, it is recommended to avoid fosfomycin as the sole antimicrobial compound for the treatment of *K. pneumoniae* infections [56]. Options for a combination antimicrobial treatment of colistin-resistant *K. pneumoniae* related infections include carbapenems, aminoglycosides and tigecycline [84].

An observational study evaluated the outcome of 41 intensive-care patients treated with intravenous fosfomycin for infections due to MDR *K. pneumoniae*, 23 of them were monomicrobial MDR *K. pneumoniae* infection. These patients received fosfomycin mainly in combination with colistin or tigecycline. The study reported a relatively high rate of successful clinical outcome, defined as the resolution of all signs of infection, in 54.2% of patients at day 14. The subgroup analysis of the 23 cases of monomicrobial *K. pneumoniae* infection reported a 56.5% rate of successful clinical outcome at day 14. Of note, fosfomycin resistance developed in three cases during the study [84].

### 2.2. Tigecycline

Tigecycline is a broad-spectrum semisynthetic glycylcycline originally derived from minocycline. Tigecycline acts inhibiting bacterial protein synthesis [61,85]. More precisely, tigecycline reversibly binds the ribosome 30S subunit interfering with the accommodation of aminoacyl-tRNA in the A site of the 16S rRNA [61,86].

Tigecycline possesses activity against Gram-positive and Gram-negative aerobes [61]. In a large antimicrobial surveillance program, the SENTRY study, performed worldwide between 2000 and 2005, tigecycline resulted generally active against the *K. pneumoniae* isolates collected in the study, confirming to be a valid option for the treatment of infections caused by *K. pneumoniae* [87].

However, as a matter of concern a multi-center, prospective cohort study on 287 hospitalized patients in the US recently reported up to 46% of tigecycline resistance among carbapenem-resistant *K. pneumoniae* isolates [88].

Recently, a retrospective cohort study examined the outcomes of 50 patients with severe infection caused by C-C-RKp [89]. The study was conducted between 2012 and 2013 in Spain, during an outbreak of MDR *K. pneumoniae*. The commonest types of infection were pneumonia and urinary tract infections, and around one-third of patients had bacteraemia. Tigecycline was active against 72% of the isolates. Combination therapy with tigecycline and gentamicin resulted to be associated with lower mortality rates (Hazard Ratio 0.37; 95% CI: 0.13–1.03; *p* = 0.058).

So far, tigecycline has been approved in Europe for the treatment of complicated skin infections and complicated intra-abdominal infections, and in the US, it has also been approved for community-acquired bacterial pneumonia [90,91]. The suggested intravenous dosage of tigecycline is an initial loading dose of 100 mg, followed by 50 mg every 12 h; in selected cases (i.e., serious systemic infections), a loading dose of 200 mg followed by 100 mg every 12 h can be advisable [63].

Tigecycline possesses interesting pharmacokinetic properties, with a long elimination half-life and a rapid systemic distribution after administration [62,92]. Of note, tigecycline shows a good distribution in the gall bladder, bile and colon after a single intravenous dose of 100 mg [63]. However, tigecycline does not possess a favorable PK/PD profile for some other sites, like bloodstream infections and urinary tract infections [93].

### 2.3. Aminoglycosides

Aminoglycosides are considered a valuable option in combination with other antimicrobials against MDR *K. pneumoniae*, if in vitro susceptibility is confirmed. In a retrospective cohort study on 50 patients with severe infection caused by C-C-RKp, combination therapy including tigecycline and gentamicin resulted to be associated with lower mortality rates [89].

Aminoglycosides should preferably be administered once daily at a dosage of 5 mg/kg and 15 mg/Kg for gentamicin and amikacin, respectively [94]. Before the establishment of therapy with aminoglycoside, clinicians should know the aminoglycosides’ MIC of the isolate. An area under the curve (AUC)/MIC ratio of 30–50 should be the target in noncritical patients, while higher AUC/MIC ratios, up to 80–100, should be the target in critical patients (i.e., immunodepressed patients, inadequate infection source control, presence of infections with a high bacterial burden) [94].

Of importance, beside the use of the AUC/MIC ratio PK/PD index to ensure efficacy, the clinical use of aminoglycosides should take into account the risk of adverse events such as nephrotoxicity and ototoxicity, and the scarce penetration into several sites such as lungs, skin and soft tissues, bone, brain, and bloodstream. In the presence of infections in these sites, antimicrobial combination non-including aminoglycosides should be favored instead of increasing the aminoglycoside dosage. Moreover, at present, the exact AUC threshold to prevent nephrotoxicity for patients receiving once daily aminoglycoside dosing is not ascertained. Therefore, if gentamicin is used, trough concentration should be monitored and maintained below 0.5–1 μg/mL to reduce the risk of nephrotoxicity [94,95].

### 2.4. Ceftazidime/Avibactam

Ceftazidime/avibactam is a β-lactam antimicrobial/β-lactamase inhibitor combination, recently approved for the treatment of abdominal infections, urinary tract infections and hospital acquired pneumonia [3,64,96,97]. The synthetic β-lactamase inhibitor avibactam inhibits Ambler class A, C and D β-lactameses, including AmpC, *K. pneumoniae* carbapenemase (KPC) and OXA-48 [64,98,99]. Ceftazidime/avibactam is bactericidal, time-dependent and is renally excreted [3,64].

Structurally, avibactam is a [1–3]-diazabicyclooctanone derivative and differs from other β-lactamase inhibitors, such as clavulanic acid, sulbactam and tazobactam [65]. In the majority of clinical trials performed so far, ceftazidime/avibactam was administered at the dose of 2.0/0.5 g every 8 h.

The avibactam β-lactamase inhibition mechanism involves a covalent binding with the β-lactamase. Interestingly, the binding between avibactam and the β-lactamase is reversible but not susceptible to hydrolysis, preventing the regeneration of the active β-lactamase enzyme observed with other β-lactamase inhibitors [65]. Avibactam expands ceftazidime spectrum of activity to include many carbapenem-resistant Enterobacteriaceae. This compound has therefore an important role in the treatment of MDR *K. pneumoniae*, including C-C-RKp [100].

Importantly, avibactam is not active against Ambler class B metallo-β-lactamases (e.g., New Delhi metallo-beta-lactamase (NDM), Verona integron-borne metallo-β-lactamase (VIM), Imipenemase (IMP)) due to the absence of the active-site serine residue in these enzymes. [66]. Interestingly, the use of ceftazidime/avibactam in combination with aztreonam has been recently proposed [66]. The rationale for this regimen is to combine ceftazidime/avibactam activity against class A and D carbapenemases with aztreonam activity against class B metallo-β-lactamases, obtaining efficacy against any *K. pneumoniae* isolate [66,101].

From a clinical point of view, the peculiar inhibitory profile of ceftazidime/avibactam against OXA-48 like carbapenemases makes this combination the first choice for MDR *K. pneumoniae* OXA-48 producer strains [102]. Ceftazidime/avibactam also represents an interesting choice in the treatment of hospital-acquired *K. pneumoniae* pneumonia that presents limited therapeutic options. At the same time, ceftazidime/avibactam is associated to a much lower risk to develop acute kidney injury during treatment in comparison with aminoglycosides or colistin.

However, as a matter of concern, Shields et al. observed the development of ceftazidime/avibactam resistance in three patients after ceftazidime/avibactam treatment, due to plasmid-borne mutations [103]. Also, Giddins et al. recently reported the development of ceftazidime/avibactam resistance in a *K. pneumoniae* clone during ceftazidime-avibactam treatment [104]. Apparently, resistance to ceftazidime/avibactam can emerge on therapy following short periods of treatment and may be also fueled by polymyxin B and carbapenem exposure [104,105,106].

Resistance mechanisms to ceftazidime/avibactam include mutations in the blaKPC gene, with KPC-2 and KPC-3 producing *K. pneumoniae* strains having higher MIC than other strains [103,104,107]. However also other resistance mechanisms involving mutation in the “OmpK36” porin may play a role [107]. The emergence of ceftazidime/avibactam resistance represents a global public health threat and warrants the implementation of active surveillance systems and infection prevention and control measures. As suggested by the European Centre for Disease Prevention and Control, even a single resistant isolate should be timely detected and reported. Microbiology laboratories have a central role in detecting and reporting resistance. It is, therefore, essential that reference microbiology laboratories are able to perform ceftazidime/avibactam susceptibility testing by appropriate phenotypic and genetic methods in clinical and screening samples [108]. Microbiological samples should be obtained prior to any ceftazidime/avibactam therapy, to inform the clinicians allowing to decide on continuation or de-escalation or escalation of an empiric therapy. [108].

## 3. Novel Antimicrobial Agents for the Treatment of C-C-RKp

### 3.1. Plazomicin

Plazomicin is a novel semisynthetic aminoglycoside derived from sisomicin [69,109,110]. Similarly, to other aminoglycosides, plazomicin acts inhibiting protein synthesis by binding to the bacterial ribosomal 30S subunit. This antimicrobial is specifically designed to be active against bacteria containing aminoglycoside-modifying enzymes such as acetyl-, phosphoryl- and nucleotidyl-transferases [67,69,109,110].

Plazomicin is therefore active against clinical isolates possessing a broad range of resistance mechanisms, including carbapenemases [111,112]. However, plazomicin is not active against many of the New Delhi metallo-β-lactamases producing isolates due to the co-production of 16S ribosomal RNA methyltransferase [111,112,113]. In an in vitro study, plazomicin activity was tested against 50 carbapenem-resistant *K. pneumoniae* strains, reporting a plazomicin MIC range between 0.125 and 1 mg/L [114]. Another recent, large study conducted in 70 US hospitals reported similar results with excellent activity [115].

However, in a large susceptibility study involving 29 hospitals in eight countries, 50 colistin-resistant *K. pneumoniae* isolates were tested against plazomicin, and 5 (10%) *K. pneumoniae* isolates resulted resistant to plazomicin with a MIC higher than 128 mg/L [109].

Regarding plazomicin adverse events, caution is required for the risk of nephrotoxicity. In patients with reduced kidney function (creatinine clearance < 90 mL/min), it is recommended to maintain plasma trough concentrations lower than 3 μg/mL [68]. From a clinical point of view, at the light of the preliminary evidences coming from the ongoing CARE study, plazomicin will probably become the mainstay of antimicrobial combination regimens against life treating *K. pneumoniae* infections, favoured by its efficacy and its better safety profile than colistin [69,116].

### 3.2. Cefiderocol

Cefiderocol is a novel “siderophore” cephalosporin [70,71,117]. This antimicrobial uses a unique “trojan horse” strategy to penetrate into the bacterial cell. Cefiderocol binds free iron ions in the extracellular space and is subsequently transported through the bacterial membrane by the iron transport system of the bacterium itself. Once inside the bacterial cell, cefiderocol binds to penicillin-binding proteins impairing the bacterial cell wall synthesis [70,71,117,118].

Cefiderocol has high stability against carbapenemase hydrolysis [69]. In vitro studies have confirmed cefiderocol activity against most Enterobacteriaceae, including KPC and Metallo-β-lactamases producer isolates, with MIC ranging from 0.125 to 4 mg/L [70,117,118]. Recently, a large evaluation of the antimicrobial activity of cefiderocol against a panel of multidrug-resistant bacterial isolates from human clinical sources showed an optimal susceptibility profile also for colistin-resistant strains (MIC90 ≤ 2 μg/mL) [72].

This novel siderophore cephalosporin appears as one of the most promising new antibiotics against colistin-resistant *K. pneumoniae*. Currently, phase III clinical trials evaluating cefiderocol for Gram-negative related urinary tract infections, bloodstream infections and hospital-acquired pneumonia are ongoing.

### 3.3. Meropenem/Vaborbactam

Meropenem-vaborbactam is a recently introduced compound, constituted by a carbapenem/β-lactamase inhibitor combination [102,119]. Vaborbactam does not possess antimicrobial activity by itself; it was designed to protect the antimicrobial meropenem from carbapenemases catalytic activity [120]. This β-lactamase inhibitor directly binds the β-lactamase catalytic serine residues and is active against class A serine carbapenemases (i.e., KPC) and class C carbapenemases (i.e., AmpC) [73,120,121]. Meropenem/vaborbactam activity was confirmed against large collection of *K. pneumoniae* [122,123]. Importantly, vaborbactam does not confer any additional efficacy against class B carbapenemases, (i.e., metallo-β-lactamases), and class D carbapenemases (i.e., OXA-48-like carbapenemases).

Regarding meropenem/vaborbactam PK/PD profile, as vaborbactam does not undergo hepatic metabolism, no dose adjustment is recommended in patients with reduced hepatic function, while dose should be reduced in presence of reduced renal function (estimated glomerular filtration rate < 50 mL/min/1.73 m^2^) [124,125,126].

At present, meropenem-vaborbactam is approved by FDA as an antimicrobial combination at a fixed-dose of 4 g (2 g meropenem/2 g vaborbactam) every 8 h as a 3-h intravenous infusion for the treatment of complicated urinary tract infection, intra-abdominal infection, hospital-acquired pneumonia including ventilator associated pneumonia and bacteraemia [74,75].

Meropenem/vaborbactam could represent a future valid option for the treatment of hospital-acquired or ventilator-associated pneumonia due to MDR *K. pneumoniae* [127]. On this issue, the phase III, double-blind, randomized “TANGO III” trial is ongoing to specifically compare efficacy and safety of meropenem-vaborbactam versus piperacillin/tazobactam for hospital-acquired and ventilator-associated bacterial pneumonia [128].

In our opinion, considering its elevated cost and the risk of resistance development, meropenem-vaborbactam should be reserved for the treatment of selected cases of infections, including C-C-RKp ones [73]. When possible, all the C-C-RKp isolates should be routinely tested against both ceftazidime/avibactam and meropenem/vaborbactam. In the clinical practice, before the start of a treatment against *K. pneumoniae*, the clinician has to keep in mind that vaborbactam is not able to restore the activity of meropenem against OXA-48-like carbapenemases and that both ceftazidime/avibactam and meropenem/vaborbactam are ineffective against metallo-β-lactamases such as NDM-1, VIM and IMP [129].

Regarding the issue of resistance development, vaborbactam crosses the outer membrane of Gram-negative bacteria using the “OmpK35” and “OmpK36” porin system and increased meropenem/vaborbactam MICs due to porins mutations have been already reported [73].

Interestingly, resistance to meropenem/vaborbactam is not affected by KPC production, while is affected by “OmpK36” porin mutations. These mutations may result either in a modification of the porin tridimensional structure with a narrower inner channel or in a decreased porin expression [130]. Currently, it is not clear if K. *pneumoniae* isolates harboring “OmpK36” mutations can be effectively treated with meropenem/vaborbactam. Therefore, whenever a treatment with meropenem/vaborbactam is considered, it is important to assess meropenem/vaborbactam susceptibility by disk diffusion methods. If possible, the underlying mechanisms of resistance should be determined [130].

### 3.4. Eravacycline

Eravacycline is a novel fully synthetic tetracycline, with a chemical structure similar to tigecycline [76,78]. Eravacycline inhibits the bacterial protein synthesis by direct binding to the bacterial ribosome [78]. However, eravacycline is not affected by the most common tetracycline resistance mechanisms (i.e., ribosomal protection proteins, β-lactamases and efflux pumps) [78,131].

Eravacycline is associated with minor adverse events including nausea and infusion site effects such as superficial phlebitis [77].

Considering the optimal distribution of eravacycline in the lung and pulmonary surfactant, this antimicrobial could represent a future option for the treatment of hospital-acquired, difficult-to-treat pneumonia [132,133].

Moreover, a unique characteristic of eravacycline in comparison to other novel antimicrobials treated in this review is its potential of oral administration. This property could allow switching from intravenous to oral administration as soon as the patient improves [133].

Interestingly, unlike other novel antimicrobial such as meropenem/varbobactam or ceftazidime/avibactam, eravacycline showed excellent activity also against *C. difficile* [134]. Thus, this medication can be considered in patients at high risk to develop *C. difficile* associated diarrhea or in patients with co-existing MDR pathogens’ and *C. difficile* infection. Of note, no reports are available on the use of eravacycline against C-C-RKp infections.

Alarmingly, in a recent study collecting clinical isolates from different regions in China, resistance to eravacycline was reported in 67/393 (17%) of *K. pneumoniae* clinical isolates. The study identified the overexpression of efflux pumps and of the transcriptional regulator RamA as the mechanism causing eravacycline resistance [135].

## 4. Non-Traditional Approaches against MDR *K. pneumoniae* Future Potential Strategies

In the future, novel therapeutic strategies will be developed against MDR pathogens, including C-C-RKp. The most promising approaches are represented by the use of bacteriophages, the administration of monoclonal antibodies targeting specific bacteria species, the genetic editing and the fecal microbiota transplantation.

Bacteriophages are bacterial predators, normally found in nature. Phage therapy was used since the 1920s in the Soviet Union, and is currently being considered as a potential future alternative to antibiotics [136,137,138]. Studies on bacteriophages against *K. pneumoniae* in animal models reported promising results [139,140]. However, crucial issues must be resolved before the implementation of the bacteriophage strategy against bacterial infections, such as the risk of a rapid occurrence of phage resistance, the risk of patient immune response to the administered phages and the lack of ad hoc regulatory requirements [136,137,138].

Monoclonal antibodies present the advantage to bind to highly specific bacterial targets, and this peculiar mechanism of action confers appealing features [141]. Monoclonal antibodies do not cause disruption of the gut flora; therefore, differently from traditional broad-spectrum antimicrobials, do not contribute to gut dysbiosis and to develop *C. difficile* infection [141,142]. Moreover, these compounds do not cause antibiotic resistance [141].

Monoclonal antibody targets are mostly represented by bacteria proteins (i.e., lipopolysaccharide), capsular polysaccharide or exopolysaccharides, pilus formation proteins and extracellular vesicle components [141]. Monoclonal antibodies targeting bacterial pili may represent a promising future approach to treat urinary tract infections due to *K. pneumoniae*, reducing bacterial adhesion and biofilm formation in the urinary tract [141].

It is likely that in the future, monoclonal antibodies will be used in combination with antimicrobials, improving treatment efficacy against MDR bacteria [141]. Interestingly, monoclonal antibodies may be administered to reduce the MDR *K. pneumoniae* gut colonization, as documented on murine experiments [143]. Currently, one major issue for the implementation of monoclonal antibodies against bacterial infections is the lack of accurate models of human infection for preclinical studies [137,141,144].

Bacterial gene editing represents another innovative tool to fight MDR bacteria (i.e., the genetic editing by the “clustered regularly interspaced short palindromic repeats” system (CRISPR/Cas)) to eliminate the gene or the genes conferring bacterial antimicrobial resistance [145].

Finally, faecal microbiota transplantation (FMT) represents a promising approach to achieve MDR Enterobacteriaceae gut decolonization [146,147,148]. This approach aims to replace the patient dysfunctional microbiota, containing MDR bacteria, with a healthy donor microbiota, without drug-resistant bacteria.

FMT seems effective especially in selected settings, such as colonised patients in haematologic wards, reducing the risk developing difficult-to-treat, life-treating infections by MDR pathogens, and reducing the burden of MDR Enterobacteriaceae in these facilities [149]. In a single-center, prospective study, 20 patients with blood disorders underwent 25 FMTs. Patients in the study were colonised by MDR Enterobacteriaceae, mostly carbapenem-resistant *K. pneumoniae*. FMT achieved *K. pneumoniae* eradication in 10/19 cases (53%) [149]. However, the FMT approach still presents unsolved questions; above all, we do not yet know how FMT exactly works.

Interestingly, Bilinski et al. found that eradication of *K. pneumoniae* may depend on the abundance of other bacterial species such as Barnesiella and Bacteroides in the transplanted fecal material [149]. Also, the potential risk of infections transmitted via FMT as well as the long-term outcomes of patients undergoing FMT are current issues. In order to mitigate these risks, meticulous donor screening has been adopted so far, including the check for the absence of viruses and other pathogens in the donor microbiota. At present, live microbiota preparations are in development to obtain the beneficial effect of FMT with a more controlled and regulated product [150].

## 5. Conclusions

The prevalence of colistin-resistant *K. pneumoniae* is increasing globally, representing a major concern, as colistin is one of the few remaining treatment alternatives for patients infected with MDR *K. pneumoniae*. Current available antimicrobial options include old antibiotics with limited PK/PD profiles under a clinical perspective, and newest ones, like ceftazidime/avibactam for which already resistance has been reported. Novel antibiotics not yet commercially available, such as plazomicin, siderophors, eravacycline and meropenem/vaborbactam, will strengthen the armamentarium against colistin-resistant *K. pneumoniae.* The recent reports on resistance to the antimicrobials treated in this review are alarming, especially for ceftazidime/avibactam, meropenem/vaborbactam and eravacycline. Carriers of bacteria resistant to these antimicrobials represent reservoirs for the transmission to other patients within the same healthcare setting or to other healthcare settings. It is of crucial importance that microbiology laboratories perform susceptibility testing for these new antimicrobials by phenotypic and genetic/molecular methods, in order to allow the implementation of surveillance programs, to permit the early identification of the carriers, to prompt timely infection control procedures, and to guide the clinicians in the choose of the appropriate therapy [108]. Furthermore, innovative approaches are in development. Nonantimicrobial compounds such as monoclonal antibodies and bacteriophages will be available to reinforce the antimicrobial treatment. Bacteriophages therapy is currently considered as a potential future alternative to antibiotics but crucial issues must be resolved before its implementation, such as the risk of a rapid occurrence of phage resistance, the risk of patient immune response to the administered phages and the need of ad hoc regulatory requirements. FMT will be probably confirmed as a valuable tool in selected settings and patients to restore microbiota colonized by MDR pathogens including C-C-RKp, thus preventing subsequent systemic infections.

## Figures and Tables

**Table 1:** Antimicrobials for C-C-RKp infections.

Antimicrobial	Antibiotic Class	Dosage	Route of Administration	Mechanism of Action	Main Feature, Advantages and Disadvantages
Fosfomycin	*Streptomyces fradiae* coltures derived antimicrobial [56]	4 g every 6 h (24 g administered daily) or more. If creatinine clearance < 30 mL/min, dosage should be reduced to 4 g every 8 h [57,58]	IV (disodic fosfomycin)	Impairs the formation of N-acetylmuramic acid, leading to bacterial death [59]	Excellent safety and tolerability profile. Excellent distribution in body sites. Monotherapy may select resistance. Requires monitoring of serum elettrolytes. An open issue is the in vitro sensibility assay interpretation [56,57,60]
Tigecycline	Semisynthetic glycylcycline [61]	Loading dose of 100 mg, followed by 50 mg every 12 h; in selected cases (i.e., serious systemic infections), a loading dose of 200 mg followed by 100 mg every 12 h can be advisable [62]	IV	Tigecycline reversibly binds the ribosome 30S subunit, inhibiting bacterial protein synthesis [61]	Good distribution in gall bladder, bile and colon. Approved for treatment of complicated skin and intra-abdominal infections [61,63]
Ceftazidime/avibactam	Third generation cephalosporin and β-lactamase inhibitor combination [64]	2.5 g every 8 h, dosage must be reduced if reduced creatinine clarance [65]	IV	Avibactam covalent binding to the bacterial β-lactamase [65]	Bactericidal Not active against class B metallo-β-lactamases [66]
Plazomicin	Semisynthetic aminoglycoside [67]	15 mg/kg every 24 h. Dosage to be reduced to 10 mg/kg every 24 h if creatinine clarance < 60 mL/min [68]	IV	Plazomicin binds the ribosome 30S subunit, inhibiting bacterial protein synthesis [67]	Active against most bacteria containing aminoglycoside-modifying enzymes. Not active against many of the New Delhi metallo-β-lactamases [69]
Cefiderocol	Siderophore cephalosporin [70]	Multiple doses of cefiderocol up to 2 g are well tolerated and exhibit linear pharmacokinetics [71]	IV	Cefiderocol binds to free iron and is transported into bacterial cells by the iron-transport system [70]	Active against most MDR isolates, including metallo-β-lactamases [72]
Meropenem/vaborbactam	Carbapenem and β-lactamase inhibitor combination [73]	4 g (2 g meropenem/2 g vaborbactam) every 8 h as a 3-h infusion. Dose adjustement if reduced creatinine clarance [74,75]	IV	Vaborbactam inhibits the target β-lactamases by directly binding to their catalytic serine residues [73]	Inactive against OXA-48-like lactamases and metallo-β-lactamases [73]
Eravacycline	Synthetic tigecycline analogue [76]	1 mg/kg by intravenous infusion over 60 min every 12 h for 4 to 14 days. Dose reduction if severe hepatic impairment and if concomitant cytochrome P450 inducer [77]	IV, oral	Inhibition of the bacterial protein synthesis by direct binding to bacterial ribosome [76,78]	Active against KPC, OXA-48-like and metallo-β-lactamases producing strains. Not effective against *P. aeruginosa* [78]

C-C-RKp = Colistin-, Carbapenem-resistant *K. pneumoniae*; KPC: *K. pneumoniae* carbapenemase. IV: intravenous.

**Table 2:** Possible antimicrobial combination therapy for C-C-RKp infections, according to the meropenem MIC value and the site of infection. The choice of antimicrobials depends on in vitro susceptibility assays.

Site of Infection	Serine Carbapenemases Producer Strain (i.e., KPC, OXA-48 Like)		Metallo-β-Lactamase Producer Strain (i.e., VIM, IMP, NDM)
	Meropenem MIC ≤ 16 mg/L	Meropenem MIC > 16 mg/L	
Bloodstream infections	ceftazidime/avibactam meropenem double dosage (prolonged infusion) + fosfomycin meropenem double dosage (prolonged infusion) + gentamicin meropenem double dosage (prolonged infusion) + fosfomycin + gentamicin	ceftazidime/avibactam ceftazidime/avibactam ± fosfomycin or gentamicin Consider fosfomycin plus gentamicin in case of resistance to ceftazidime/avibactam Future options: cefiderocol plazomicin meropenem/vaborbactam (not active against OXA-48-like carbapenemases)	ceftazidime/avibactam + aztreonam Future option: cefiderocol
Hospital acquired pneumonia, including VAP	meropenem double dosage (prolonged infusion) + fosfomycin ceftazidime/avibactam ± fosfomycin ± gentamicin	ceftazidime/avibactam + fosfomycin ± gentamicin Consider fosfomycin plus gentamicin in case of resistance to ceftazidime/avibactam Future options: meropenem/vaborbactam (not active against OXA-48-like carbapenemases)	ceftazidime/avibactam + aztreonam Future option: cefiderocol eravacycline
Abdominal infections	ceftazidime/avibactam + tigecycline ± gentamicin meropenem double dosage (prolonged infusion) + tigecycline ± gentamicin	ceftazidime/avibactam + tigecycline ± gentamicin ceftazidime/avibactam + tigecycline ± fosfomycin Future options: plazomicin meropenem/vaborbactam (not active against OXA-48-like carbapenemases)	ceftazidime/avibactam + aztreonam Future option: cefiderocol
Urinary tract infections	ceftazidime/avibactam ± fosfomycin ± gentamicin meropenem double dosage (prolonged infusion) ± fosfomycin ± gentamicin consider fosfomycin trometamol for uncomplicated urinary tract infections	ceftazidime/avibactam ± fosfomycin ± gentamicin consider fosfomycin + gentamicin in case of resistance to ceftazidime/avibactam Future options: meropenem/vaborbactam (not active against OXA 48-like carbapenemases)	ceftazidime/avibactam + aztreonam Future option: cefiderocol plazomicin
Complicated skin and skin structure infections	meropenem double dosage (prolonged infusion) ± tigecycline ceftazidime/avibactam ± tigecycline	ceftazidime/avibactam ± tigecycline ceftazidime/avibactam ± fosfomycin ceftazidime/avibactam + tigecycline ± fosfomycin	ceftazidime/avibactam + aztreonam Future option: cefiderocol

Source control is recommended within 24 h of the diagnosis of intra-abdominal infection to remove infected fluid and tissue and to prevent ongoing contamination. C-C-RKp = Colistin-, Carbapenem-resistant *K. pneumoniae*; KPC: *K. pneumoniae* carbapenemase; VIM: Verona integrin encoded metallo-β-lactamase; IMP: Imipenemase; NDM: New Delhi metallo-β-lactamase; VAP: Ventilator associated pneumonia.
